# A Multimodal Magnetic Resonance Imaging Study on Myalgic Encephalomyelitis/Chronic Fatigue Syndrome: Feasibility and Clinical Correlation

**DOI:** 10.3390/medicina60081370

**Published:** 2024-08-22

**Authors:** Raminder Kaur, Brian Greeley, Alexander Ciok, Kashish Mehta, Melody Tsai, Hilary Robertson, Kati Debelic, Lan Xin Zhang, Todd Nelson, Travis Boulter, William Siu, Luis Nacul, Xiaowei Song

**Affiliations:** 1 Research and Evaluation, Fraser Health Authority, Surrey, BC V3T 0H1, Canada; raminder.kaur1@fraserhealth.ca (R.K.); brian.greeley@fraserhealth.ca (B.G.); aciok@sfu.ca (A.C.); kashish.mehta@cw.bc.ca (K.M.); todd_nelson@sfu.ca (T.N.); 2 Biomedical Physiology & Kinesiology, Simon Fraser University, Burnaby, BC V5A 1S6, Canada; 3 Women’s Health Research Institute, Vancouver, BC V6H 3N1, Canada; 4 Complex Chronic Diseases Program, BC Women’s Hospital, Vancouver, BC V6H 3N1, Canada; travis.boulter@phsa.ca; 5 ME/FM Society of BC, Vancouver, BC V6J 5M4, Canada; 6 Medical Imaging, Royal Columbian Hospital, New Westminster, BC V3L 3W7, Canada; william.siu@fraserhealth.ca; 7 Department of Family Practice, University of British Columbia, Vancouver, BC V6T 1Z3, Canada

**Keywords:** myalgic encephalomyelitis/chronic fatigue syndrome (ME/CFS), brain function, brain metabolites, single-voxel magnetic resonance spectroscopy (SV-MRS), task-phase functional magnetic resonance imaging (fMRI)

## Abstract

*Background/Objectives*: Myalgic encephalomyelitis/chronic fatigue syndrome (ME/CFS) is a neurological disorder characterized by post-exertional malaise. Despite its clinical relevance, the disease mechanisms of ME/CFS are not fully understood. The previous studies targeting brain function or metabolites have been inconclusive in understanding ME/CFS complexity. We combined single-voxel magnetic resonance spectroscopy (SV-MRS) and functional magnetic resonance imaging (fMRI). Our objectives were to examine the feasibility of the multimodal MRI protocol, identify possible differences between ME/CFS and healthy controls (HCs), and relate MRI findings with clinical symptoms. *Methods*: We enrolled 18 female ME/CFS participants (mean age: 39.7 ± 12.0 years) and five HCs (mean age: 45.6 ± 14.5 years). SV-MRS spectra were acquired from three voxels of interest: the anterior cingulate gyrus (ACC), brainstem (BS), and left dorsolateral prefrontal cortex (L-DLPFC). Whole-brain fMRI used n-back task testing working memory and executive function. The feasibility was assessed as protocol completion rate and time. Group differences in brain metabolites and fMRI activation between ME/CFS and HCs were compared and correlated with behavioral and symptom severity measurements. *Results*: The completion rate was 100% regardless of participant group without causing immediate fatigue. ME/CFS appeared to show a higher N-Acetylaspartate in L-DLPFC compared to HCs (OR = 8.49, *p* = 0.040), correlating with poorer fatigue, pain, and sleep quality scores (*p*’s = 0.001–0.015). An increase in brain activation involving the frontal lobe and the brainstem was observed in ME/CFS compared to HCs (Z > 3.4, *p*’s < 0.010). *Conclusions*: The study demonstrates the feasibility of combining MRS and fMRI to capture neurochemical and neurophysiological features of ME/CFS in female participants. Further research with larger cohorts of more representative sampling and follow-ups is needed to validate these apparent differences between ME/CFS and HCs.

## 1. Introduction

Myalgic encephalomyelitis/chronic fatigue syndrome (ME/CFS) is a complex neurological disorder characterized by persistent and debilitating fatigue associated with widespread neuroinflammation and immune system dysfunction [1,2,3]. ME/CFS impacts approximately 1% of the population globally, primarily women, including over four hundred thousand Canadians [4,5,6]. The symptoms of ME/CFS include fatigue, impaired cognition, psychomotor slowing, headache, pain, and severe dysautonomia, all of which negatively and substantially impact on one’s quality of life [1,7,8]. Despite its clinical relevance, ME/CFS has long been poorly understood, limiting effective treatment [2,8,9]. Due to its neurological symptoms, there is a need to better understand what happens in the brain underlying the known clinical complexity and heterogeneity of ME/CFS [3,9,10].

Magnetic resonance imaging (MRI) techniques have been employed to study brain metabolites (using proton ^1^H magnetic resonance spectroscopy (MRS)) or activity (using functional MRI-fMRI) in ME/CFS [11,12,13,14,15]. MRS quantifies the concentration of major metabolites by analyzing their resonate frequencies [16,17]. The most studied neuronal metabolites include N-acetyl aspartate (NAA), a marker for neuronal viability; creatine (Cr), essential for neuronal energy; phosphocholine (PCh), involved in membrane synthesis; myo-inositol (Ins), necessary for membrane function; and glutathione (GSH), a marker for oxidative stress. MRS imaging allows for the simultaneous acquisition of data from larger areas, whereas single voxel MRS (SV-MRS) focuses on a localized voxel of interest (VOI) with a higher signal-to-noise ratio [16,17,18]. fMRI detects variations in blood oxygen level-dependent signals associated with brain activity to infer functional activation [19,20]. During task-phase fMRI, a task such as the n-back is employed (a continuous performance test in which a sequence of stimuli is presented to the participants to determine if the current stimulus matches the ones shown “n” stimuli earlier in the sequence) that engages working memory and executive function to assess regional brain activity [19,20]. 

Previous MRS imaging studies have suggested widespread metabolite abnormalities in ME/CFS compared to HCs, while SV-MRS revealed lowered Cr and GSH levels in the anterior cingulate gyrus (ACC) [14,15]. In addition, a reduced NAA to Cr ratio in the left dorsolateral prefrontal cortex (L-DLPFC) has been correlated with an increased frequency of pain symptoms in ME/CFS [21]. The research has also suggested the critical role of the brainstem (BS) in ME/CFS, linked to its vital life functions and connections that often show impairments in ME/CFS [22]. Previous fMRI studies have reported greater activation in several regions of the prefrontal cortex during an easy working memory task, as well as widespread whole-brain activation during more demanding test conditions in ME/CFS, compared to HCs [11,23,24]. 

Despite increasing research efforts, MRI findings on ME/CFS remain inconclusive. Combining MRS and fMRI techniques can allow for neurochemical quantification and neuro-hemodynamic investigations within the same session. This can provide insights linking brain metabolites and functional activity to a potentially better understanding of ME/CFS. Here, we conducted a study that combines SV-MRS and fMRI techniques together with other behavioral and clinical measures. However, a multimodal MRI protocol can be challenging for participants with ME/CFS who suffer from fatigue and pain; therefore, our first aim was to assess the feasibility of the protocol, i.e., how well ME/CFS participants completed the study. 

Our second aim was to identify possible metabolite and functional differences between the patients with ME/CFS and HCs. To assess the clinical relevance of the MRI data, we correlated SV-MRS and fMRI findings with symptom assessments (e.g., pain, fatigue). Based on the current research highlighting the significant involvement of ACC, BS, and L-DLPFC in ME/CFS, we conducted MRS measurements from these three specific brain regions. Similarly, encouraged by the positive fMRI findings applying n-back tasks, we adapted a version of the task with two levels of difficulty (i.e., 1- and 2-back conditions). 

## 2. Materials and Methods

### 2.1. Participants

This study enrolled 18 female patient participants aged 24–68 years (mean: 39.7 ± 12.0) with clinically diagnosed ME/CFS. Patients were identified through the Complex Chronic Diseases Program (CCDP) Data Registry at British Columbia Women’s Health Centre, Canada. Inclusion criteria were as follows: (i) female; (ii) aged 19–69; (iii) with ME/CFS based on the 2015 Institute of Medicine or the 2003 Canadian Consensus Criteria or both [25,26]; (iv) have symptoms of ME/CFS between 6 months to 5 years at recruitment; (v) have normal or corrected-to-normal vision and hearing. CCDP confirmed patient diagnosis by physician interview. Applying the CCDP Data Registry, researchers relied on the CCDP doctors to confirm diagnosis as per their standard clinical process, for ME/CFS patients being allowed to participate in the CCDP clinical program and the research study. If the CCDP program deemed a patient not eligible for the CCDP, they were identified as no longer eligible for the CCDP Data Registry. Confirmation of diagnosis from CCDP doctors was obtained via a patient’s clinical chart. In addition, five female control participants (HCs) aged 28–58 years (mean: 45.6 ± 14.5), each representing an age group (20s, 30s, 40s, 50s, and 60s), were recruited through word of mouth and recruitment posters. HC participants were required to have no known neurological or neuro/cardiovascular diseases. Participant enrolment was completed within three months of the recruitment initiation (i.e., 8 September–4 November 2022).

### 2.2. Study Design

**Figure 1:**
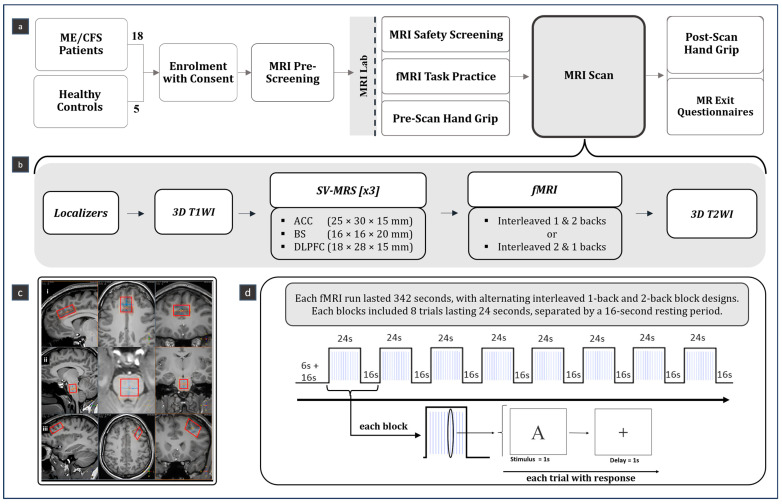
Study design of the multimodal MRI on ME/CFS project. (**a**) overview of the study design; (**b**) magnetic resonance imaging (MRI) protocol; (**c**) single voxel magnetic resonance spectroscopy (SV-MRS): red squares indicate the voxel placements in the following brain regions: (i) anterior cingulate gyrus; (ii) brainstem; (iii) left dorsolateral prefrontal cortex; (**d**) paradigm of functional MRI (fMRI) task using n-back. MR, magnetic resonance; MRI, magnetic resonance imaging; fMRI, functional MRI; MRS, magnetic resonance spectroscopy; ACC, anterior cingulate cortex; BS, brainstem; L-DLPFC, left dorsolateral prefrontal cortex; 3D, 3 dimensional; T1WI, T1-weighted imaging; T2WI, T2-weighted imaging; SV-single voxel.

The MRI scan for each participant was completed within one week after the enrolment at the SFU ImageTech Lab at the Surrey Memorial Hospital of Fraser Health, Canada. Upon MRI pre-screening approval at the lab, participants first underwent a standard MRI safety screening conducted by an MR technologist (Figure 1a). Participants were introduced to the n-back task, which includes two conditions: an easier 1-back and a more difficult 2-back (Figure 1b). Handgrip strength (HGS) was assessed to estimate the fatigue level, administered before and immediately following the MRI (Figure 1a). 

The MRI session with data collection was designed for completion within one hour. Once participants were comfortably positioned in the scanner, the scanning protocol was initiated with the localizer. The protocol included a 3-dimensional T1-weighted imaging for voxel placement and co-registration, SV-MRS for each VOI, followed by task-phase fMRI with 1- and 2-back conditions and concluded with T2-weighted imaging (Figure 1b). MRI data were acquired using a Philips Ingenia 3.0T CX clinical scanner (Philips Healthcare, Best, The Netherlands) equipped with a 32-channel dStream head coil. 

### 2.3. SV-MRS Data

SV-MRS data were acquired using a semi-LASER sequence (TR/TE = 5000/36; number of acquisitions = 64; spectral width = 2000 Hz; data points = 2048; flip angle = 90) in 3 VOIs (Figure 1c): ACC: 25 × 30 × 15 mm = 11.25 mL, BS: 16 × 16 × 20 mm = 5.12 mL, and L-DLPFC: 18 × 28 × 15 mm = 7.56 mL.

Spectra data were exported using Philips’ standard SPAR/SDAT file extensions and preprocessed using FID-A [27]. The radio frequency (RF) coil combination for signal averaging was carried out according to Near and colleagues (2021) [28]. Eddy current correction was performed by applying unsuppressed water spectra acquired for each VOI. During quality control, spectra were examined for artifacts such as poor water suppression and lipid contamination. Preprocessed metabolite data were quantified, and water was suppressed in the time domain using LCModel (Linear Combination of Model) software package (version 6.3-1R, Oakville, ON, Canada) [29].

Voxel-wise tissue segmentation was performed to obtain the percentage composition of white matter, grey matter, and cerebrospinal fluid using the Functional Magnetic Resonance Imaging of the Brain Software Library (FSL) svs_segment function [30]. Cerebrospinal fluid, which contains small concentrations of metabolites, was used to correct for the partial volume effect using the following equation [13,16]. Metabolite readings are skewed by the underlying composition of the imaged region, as follows:

$$M_{corr}=M_{raw}*\frac{1}{1-fCSF}$$

where $M_{corr}$ is the corrected metabolite concentration; $M_{raw}$ is the metabolite value output from LCModel; and fCSF is the fractional cerebrospinal fluid composition of the voxel obtained tissue segmentation.

Multiple metabolites were examined for potential quantification, including NAA, Cr, PCr, PCh, Ins, GSH, gamma-aminobutyric acid (GABA, an inhibitory neurotransmitter), glutamine (GLN, a neurotransmitter precursor amino acid), glutamate (GLU, an excitatory neurotransmitter), and N-acetyl aspartyl glutamate (NAAG, a modulator of glutamatergic neurotransmission) [18]. Metabolites were expressed in institution units. Metabolites were excluded from further analysis if the Cramér–Rao lower bounds for model fitting were >50% to ensure good quality of fitting [29].

### 2.4. fMRI Data

Blood oxygen level-dependent fMRI utilized echo planar imaging (GRE-EPI: TR/TE = 2000/30 ms, flip angle = 90°, voxel = 3 mm^3^, FOV = 24 cm^2^, with 36 axial slices covering the whole brain). The fMRI task employed a block design. The task commenced with a 16 s initial resting period, followed by eight interleaving blocks of 1-back and 2-back conditions (Figure 1d), counterbalanced among participants. Each block contained 12 trials of randomized single-letter stimuli for each participant. Each stimulus was displayed for 1s, which was followed by a 1s fixation period displaying a “+” in the middle of the screen (Figure 1d). Participants viewed the stimuli on the screen via a mirror attached to the head coil and used a response pad of two buttons using their dominant hand to indicate a match (e.g., the same consequent letter–an index finger button-press) or a non-match (e.g., different consequent letters–a middle finger button-press). Before undergoing the MRI scan, each participant practiced the task on a laptop multiple times to ensure performance was above chance and could understand and follow task instructions adequately. Task presentation and response recording were conducted using Presentation^®^ (^©^ 2023 Neurobehavioral Systems, Inc., Berkeley, CA, USA) and synchronized with the MRI via Invivo SensaVue (Philips, Gainesville, FL, USA) and Lumina 3G Controller (Cedrus Corporation, San Pedro, CA, USA). 

Standard fMRI data processing and analysis were conducted using FSL’s Feat v6.00, involving brain extraction, motion correction, spatial smoothing (6 mm), high pass filtering (100 s), and co-registration to the 3D T1WI (T1 weighted image). The MELODIC procedure was applied with a probability threshold of 0.5 for independent component analysis to remove noise sources such as sudden changes, high-frequency dominance, and numerous small activation clusters from the fMRI signal [31]. Two raters independently discerned the noise components with an intraclass correlation coefficient > 0.75 across the dataset (indicating good reliability) [32]. Individual-level data were modelled voxel-wise using the general linear model (glm) with a 5 mm smoothing kernel; threshold at Z > 2.3, *p* < 0.050, cluster-corrected, to generate activation maps for each condition (1-back and 2-back) [33]. The individual activation and contrast maps were registered to a standard space (i.e., MNI152 template) for group-level analysis, which utilized a fixed effects model with an unpaired t-test. Subject group means (ME/CFS, HCs) and contrasts (ME/CFS > HCs, HCs > ME/CFS) were computed for each task (threshold at Z > 3.4, *p* < 0.010, cluster-corrected) [34]. Activation maps were superimposed on the axial slices and rendered on 3D surfaces of standard anatomical imaging using the Montreal Neurological Institute’s template of 152 averaged brain images (MNI152) for visualization purposes. Identification of activated brain regions was based on the Harvard/Oxford cortical and subcortical structure atlas with Featquery [35].

### 2.5. Behavioral Data

Response accuracy (defined as the percentage of correctly identified matches and non-matches to all responses) and reaction time (RT, defined as the time elapsed between the onset of the target stimulus and the beginning of the button press) for the fMRI task conditions were evaluated. After confirming a normal distribution, data underwent a two-way mixed repeated measure analysis of variance (ANOVA) with group (ME/CFS, HCs) as the between-subjects factor and difficulty (1-back, 2-back) as the within-subjects factor. Accuracy comparisons between subject groups were assessed using the Mann–Whitney U, while comparisons between task conditions were evaluated using the Wilcoxon signed-ranks test. 

### 2.6. Handgrip Strength

The HGS tests were conducted pre- and post-MRI scans using the JAMAR^®^ hydraulic hand dynamometer (Performance Health, Warrenville, IL, USA). Participants were instructed to squeeze the dynamometer as firmly as possible for three seconds, followed by a 30 s rest period between measurements [36]. Three measurements were taken during each HGS test (i.e., pre- and post-MRI scan), and the mean in kilograms, standard deviation (SD), and coefficient of variance (COV, a unit free assessment of the variation) were analyzed.

### 2.7. Clinical Data

At the MRI appointment, patient-reported symptoms using self-completed questionnaires were retrieved using REDCap, a provincial-wide web-based software used for designing and capturing data for research studies, accessible at BC Women’s Health Centre. The questionnaires included Quality of Sleep (QOS), Fatigue Severity Scale (FSS), and Visual Analog Fatigue Scale (VAFS), each of which was standardized to 100 (a higher value indicates a more severe condition). In addition, the Visual Analog Pain Scale (VAPS) using the Pain and Sleep Questionnaire and the Symptoms Assessment Questionnaire, the Generalized Anxiety Disorder-2 (GAD-2), and Patient Health Questionnaire-2 (PHQ-2) scores were retrieved from REDCap (^©^ 2024 Vanderbilt University, version 14.1.2, Nasville, TX, USA).

### 2.8. Statistics

Statistical analyses on demographics, symptoms, behavior, metabolites, and fMRI activations were carried out using RStudio (version 1.4.1717), MATLAB (version 2021), and SPSS (version 26). Depending on the data distribution, independent samples t-tests or Mann–Whitney tests were used to test group features (age, grip strength pre, sleep quality, FSS, pain intensity, GAD-2, PHQ-2, and HGS). Univariate logistic regression models were run using the glm function within RStudio to identify metabolites that best differentiated the HC and ME/CFS groups within each VOI. Ten separate univariate logistic regressions were conducted for each VOI (e.g., ACC, BS, DLPFC) for each metabolite. The model outputs included odds ratio (OR), coefficients (i.e., estimates), standard error, z-value (Wald’s test or z-statistic), and the associated *p*-value. Tests for multiple simultaneous comparisons were performed based on the Bonferroni correction threshold. Differences were compared between participant groups. The Spearman correlation coefficient was used to test the correlation of the clinical symptom scores with the MRS, fMRI, behavioral, and HGS measurements that showed significant participant–group differences. A linear regression model was used to fit the correlated relationship using the least squares method. Statistical significance was set at *p*-values < 0.050 and adjusted for false discovery rate for multiple comparisons. 

## 3. Results

Regarding feasibility, in each participant group, the rate for completing the one-hour MRI protocol, was 100%. Further, the MR sequences went smoothly with each participant, i.e., in no cases was any sequence (SV-MRS of three VOIs, task-phase fMRI, and anatomical T1WI and T2WI) interrupted, needing a repeat, or incomplete.

Participants with ME/CFS showed a poorer mean score in each clinical symptom relative to HCs (Table 1). The mean handgrip strength did not differ between the ME/CFS and HC groups or change pre- and post-MR experiment in either group (*p* > 0.05). Considering measures at both time points, the patient participants were significantly weaker and more varied overall in the handgrip strength tests (Table 1). 

**Figure 2:**
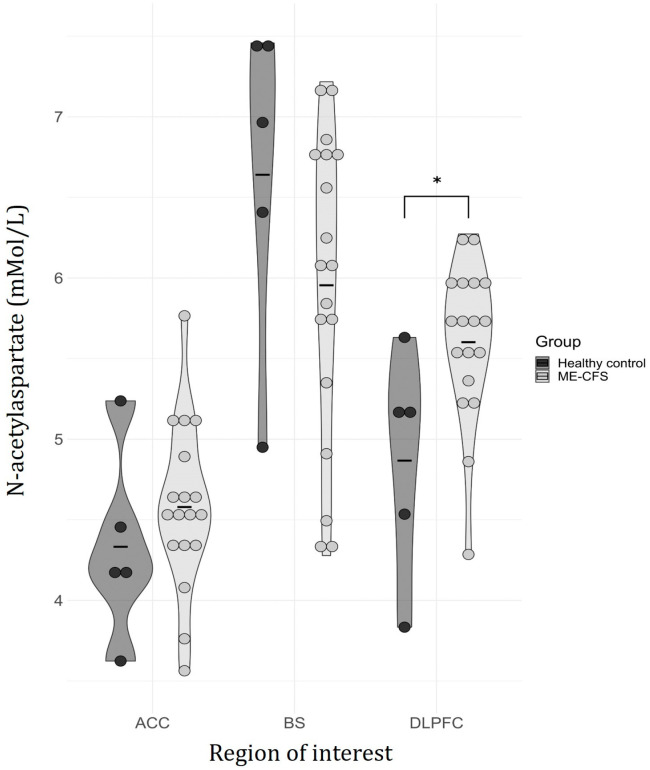
Metabolite N-acetyl aspartate (NAA) concentrations in each region of interest separated by healthy controls (HCs) (black) and ME/CFS (gray). Note: ACC, anterior cingulate cortex; BS, brainstem; DLPFC, dorsolateral prefrontal cortex; ME/CFS, myalgic encephalomyelitis/chronic fatigue syndrome. Black horizontal lines within each plot represent the mean, whereas circles represent individual data points for healthy controls (black) and individuals with ME/CFS (gray). The width of each curve corresponds to the approximate frequency of data points. * indicates a significant difference in NAA was observed in the DLPFC (OR = 8.49, *p* = 0.042, uncorrected).

A difference in the level of NAA between ME/CFS and HCs was observed within the DLPFC VOI (OR = 8.49, *p* = 0.042, which did not reach the corrected threshold of significance with multiple comparisons; Table 2), but not the other two VOIs. No statistical differences were identified in any other metabolites in any VOIs (Figure 2). 

**Figure 3:**
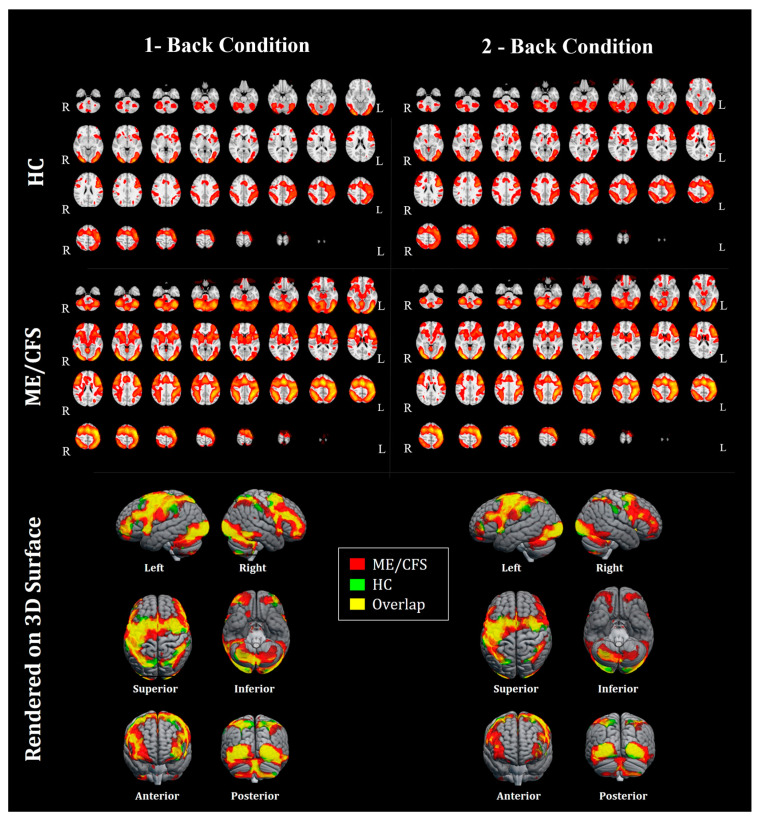
Group mean fMRI activation during the n-back task. The top and middle panels show the group mean activation maps under each condition passing the threshold (z = 3.4) for healthy controls (HCs–top panel) and myalgic encephalomyelitis/chronic fatigue syndrome (ME/CFS–middle panel). The “R” and “L” indicate the right and left side of the brain, respectively. Levels of significance are shown in red (*p* < 0.05) and yellow (*p* < 0.01), cluster-wise corrected. The bottom panel shows clusters passing the significance threshold (z = 3.4) rendered on the standard anatomical space (i.e., MNI 152). Clusters of ME/CFS (red) and HCs (green) groups, and those overlap between the groups (yellow) are displayed.

Each fMRI task condition (i.e., 1-back and 2-back) evoked distributed brain activation that involved the visual, motor, supplementary sensory, and prefrontal cortices in both ME/CFS and HC groups (Figure 3, top and middle panels). During each task condition, ME/CFS showed increased brain activation than HCs, particularly involving the frontal pole, ACC gyrus, and BS regions (Figure 3, bottom panels; Table S1). 

Associated with a comparative, less intensive fMRI action, both ME/CFS and HC participants were slower (F = 12.61; *p* < 0.001) and less accurate (Z = −2.18, *p* = 0.030) in the 2-back compared to the 1-back condition (Table 3; *p* = 0.001). No group difference was found in task accuracy or reaction time, nor a group–task interaction (Table 3).

**Figure 4:**
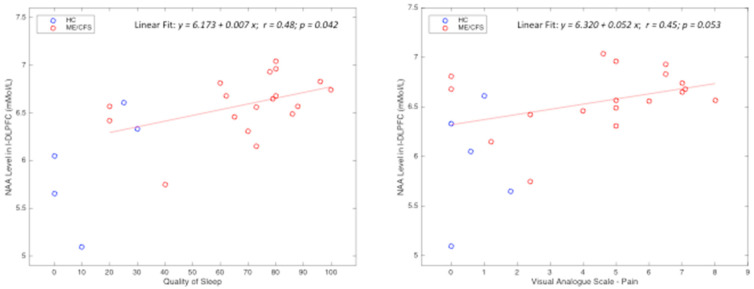
Example relationships between NAA metabolite measurements within the L-DLPFC and clinical symptoms. Note: HC, healthy control; ME/CFS, myalgic encephalomyelitis/chronic fatigue syndrome. Symbols represent observational data: red circles indicate ME/CFS participants and blue circles denote HC participants. Lines show the fitting for only the ME/CFS patient participants using linear regression. r, correlation coefficient; *p*, level of significance.

NAA in L-DLPFC correlated with several symptom severity scores assessing sleep quality, fatigue severity, and pain (r’s = 0.64–0.42, *p’s* = 0.004–0.040, false discovery rate adjusted; Table 4). Additional correlations were tested between the visual analogue scale–pain and the total number of activated voxels and the maximum Z-value of fMRI activation intensity (while the latter two were highly correlated r = 0.73; *p* < 0.001). In addition, the overall HGS was associated with worsened pain and poorer sleep quality (Table 4). Interestingly, correlations of the L-DLPFC NAA level with sleep quality and pain severity were observed within just the ME/CFS patients, suggesting a relationship independent of diagnosis (Figure 4). 

## 4. Discussion

### 4.1. Summary of Key Findings

We conducted the first multimodal MRI investigation, acquiring SV-MRS data from three brain regions implicated in ME/CFS and whole-brain fMRI data during a cognitive task, alongside clinical symptoms, grip strength, and behavior. In the female participants, we observed a trended elevation of NAA concentration limited to the L-DLPFC in ME/CFS compared to HCs. Regardless of group, NAA correlated the symptom scores on fatigue, sleep, and pain. In addition, higher fMRI activation when working memory was engaged was found in ME/CFS relative to HCs, with widespread activity including the bilateral prefrontal cortices and the brainstem. Furthermore, correlations between symptom measurements informed the clinical relevance of the brain MRI findings.

### 4.2. Interpretation

Our study demonstrated the feasibility of combining SV-MRS and fMRI studying ME/CFS. Although the ME/CFS group generally did not show consistent strength relative to HCs as measured by HGS, all participants completed the study. While post-exertional malaise (an unusual, debilitating response to even minimum levels of physical or mental exertion) is characteristic of ME/CFS, the one-hour MRI scanning protocol did not induce immediate physiological exhaustion, as measured by the HGS. As there were no follow-up evaluations in the study, it is unknown whether the scanning protocol caused fatigue in the longer term. The ME/CFS group displayed accuracy and reaction time comparable to that of the HC group when performing the n-back. This finding suggests that the conditions (including the more difficult 2-back) were not behaviorally more challenging for ME/CFS patient participants. Future work adapting the protocol should include follow-ups to better understand ME/CFS and assess the protocol’s possible longer-term effects on the condition, as in the ongoing clinical trial employing this protocol [37]. 

NAA in the L-DLPFC appeared to be higher in ME/CFS than HCs, though this novel and unexpected finding requires validation with larger sample cohorts for improved statistical power to survive the false positive adjustment of multiple comparisons. NAA is present solely in neurons and is a source of glutamate metabolism for critical neuronal and nutrient cell functions [16,38]. Within this context, stable NAA suggests that participants with ME/CFS have a functioning, relatively responsive L-DLPFC. However, considering that the ME/CFS group reported greater overall fatigue and pain compared to the HC group, it is likely that the NAA level may represent a potentially negative downstream effect of ME/CFS pathophysiology. This is supported by the observed positive correlations between the L-DLPFC NAA and the pain and sleep scores in the ME/CFS group (Figure 4). At this time, it remains unknown whether ME/CFS involves a unique metabolite profile. Given the sample size, how the metabolites in ME/CFS and HCs compare in these brain regions warrants verification in future longitudinal studies with larger cohorts and repeated measures over the course of the disorder. Further research can then target how such a profile is linked to the underlying neural immune inflammation or other hypothesized mechanisms [2,14,39,40].

Our NAA results contribute to that of the other studies investigating MRS in ME/CFS. Brooks and colleagues reported a reduced concentration of NAA in CFS (n = 7) than in age-matched controls (n = 10) [41]. Although this seems to conflict with the findings from our study, it is important to note that their data were acquired from the right hippocampal area, a subcortical brain region on the opposite hemisphere from the VOI in the current study. In a more recent study with a larger sample, Thapaliya and colleagues demonstrated a high NAA level in the posterior cingulate cortex in long COVID patients who displayed some ME/CFS-like symptoms (alongside elevated glutamate) in patient groups compared to controls [42]. The finding was interpreted as a compensatory response to brain inefficiency in patients, with increased glucose and oxygen utilization under infection-triggered osmotic stress. Similar to the current study, associations between neurochemical and clinical severity measures were found, linking neurochemistry to neuropathology, overlapping long COVID and ME/CFS [42]. Future studies comparing metabolites from the same VOIs across multiple brain regions key to ME/CFS will enhance result validation and interpretation. 

We also observed an overall greater level of fMRI activation in ME/CFS during the task that engaged working memory, despite the same level of accuracy and reaction time as HCs (Figure 3; Table S1). This finding aligns with the previous research. For instance, Shan and colleagues reviewed that across ten studies, ME/CFS participants recruited additional brain regions during a variety of cognitive tasks to achieve the same task performance as control participants [11]. This suggests individuals with ME/CFS engage the same brain regions more extensively compared to HCs, possibly associated with a compensatory mechanism to counteract impairments or dysfunction [11,43]. In addition, the increased fMRI activation observed in the BS within the ME/CFS group supports the growing evidence of BS involvement in ME/CFS [22]. Whether the region-specific functional/capacity recruitment is unique to ME/CFS is not yet known, and our study highlights the need for future research to target functional responses in these areas with the region of interest-based analysis.

### 4.3. Limitations

Several caveats apply to our study. First, the small, imbalanced sample size of female participants may restrict the sensitivity and generalizability of the results. Future studies with larger sizes of a more representative sample are warranted to verify our findings. Second, adopting an SV-MRS approach restricted location(s), we targeted three brain regions. This results in missing information in other brain regions outside of the ACC, DLPFC, and BS VOIs. Future research is needed to understand how to balance the superior signal-to-noise ratio of SV-MRS and the widespread coverage of MRS imaging in different studies. A key factor for strengthening result interpretation concerns the severity of ME/CFS in patient participants. Thus, there is likely some selection bias with participant enrolment in that less severely affected patients volunteer to participate, as was our finding, and was the case with all the MRI studies on ME/CFS. It is important to note, however, that based on fatigue severity, our patient participants were within the range of that of patients in other MRI on ME studies [14,44]. Consistent with the notion that patients with ME/CFS form a heterogeneous group, we acknowledge the individual variability among ME/CFS participants in terms of symptom expression and the lack of immediate clinical assessments at the time of the MRI scan. This could affect how we grouped participants and the sensitivity of our analysis, even though diagnosis of patient participants closely followed standard clinical procedures, and the heterogeneity of our sample was comparable with the other MRI on ME studies [10,14,18]. Understanding and addressing the variability in ME/CFS symptom expressions in future studies is crucial, and feasible physiological assessments during everyday activities can be beneficial [45]. Alternatively, previous studies have sought to enhance participant homogeneity when targeting certain ME/CFS symptoms/types through applying more rigorous selection [10,18,26,46].

### 4.4. Significance and Clinical Implication

ME/CFS is a highly impactful but largely understudied neurologic condition. The multifaceted symptom presentation in patients with ME/CFS suggests the complex origins and underlying mechanisms. While the neuropathological mechanism of ME/CFS is unclear, recent studies have targeted the immune system and neuroendocrine/metabolic abnormalities that are triggered by infection [9,18,39]. There is a pressing need for novel research and approaches toward a better understanding of the brain changes characterizing ME/CFS for potentially improving treatment and patient care [3,9,40]. Our study contributes to the field by establishing and testing the combined SV-MRS and fMRI protocol in ME/CFS research for potentially integrating brain metabolite and functional changes, demonstrating the feasibility of the protocol for future applications. Our study further pinpoints several notable alterations in the brain associated with ME/CFS, highlighting the enhanced utility of MRI in detecting clinically meaningful abnormalities and capturing underlying pathophysiological changes. Methodologically, combining SV-MRS and fMRI and comparing key brain regions in ME/CFS enhances the precision and depth of the investigation, offering insights into the neurochemical and hemodynamic aspects underlying ME/CFS. Furthermore, revealing a link between brain MRI changes and symptomatic manifestations of ME/CFS sheds light on the neurological basis of the disorder, potentially informing future clinical management. 

## 5. Conclusions

An MRI protocol integrating SV-MRS at multiple locations and task-phase whole-brain fMRI on cognition is feasible for investigating changes in brain metabolites and functional activation associated with ME/CFS symptom expression in female participants. Research building on this groundwork will extend these findings with increased sample size and more representative longitudinal cohorts. Future studies should also aim to apply comprehensive analyses to better understand the relationships between MRS and fMRI changes in brain regions critically involved in ME/CFS. 

## Figures and Tables

**Table 1:** Participant demographics comparing healthy controls (HCs) and patients with myalgic encephalomyelitis/chronic fatigue syndrome (ME/CFS).

Characteristic	HCs [n = 5]	ME/CFS [n = 18]	*p*-Value
Age	45.6 (14.5)	39.7 (12.0)	0.471
Quality of sleep	13.0 (14.0)	69.4 (22.6)	0.002 ‡
Fatigue Severity Scale	16.0 (4.9)	59.6 (5.0)	0.001 ‡
Visual Analogue Fatigue Scale	29.3 (25.3)	76.0 (9.1)	0.033
Visual Analogue Pain Scale	6.8 (7.0)	45.9 (24.2)	<0.001
Generalized Anxiety Disorder 2-item	0.0 (0.0)	1.4 (1.0)	0.004 ‡
Patient Health Questionnaire-2	0.4 (0.5)	1.8 (0.9)	0.009 ‡
Averaged hand grip strength in Kg	31.2 (6.0)	25.6 (6.7)	0.050 β
Average hand grip strength-COV	0.05 (0.03)	0.07 (0.06)	0.014

Note: Sample sizes are shown in the squared parentheses. Standard deviations are shown in brackets. ‡, denotes the use of Mann–Whitney U test; β, notes a main effect of group; Kg, kilogram; COV, coefficient of variance.

**Table 2:** Means and standard deviations (SD) and output of univariate logistic regression for each metabolite across each voxel of interest (VOI) for healthy controls (HCs) and myalgic encephalomyelitis/chronic fatigue syndrome (ME/CFS).

Voxel of Interest	Metabolite	HCs Mean (SD)	ME/CFS Mean (SD)	Odds Ratio	Z-Value	*p*-Value
**Anterior cingulate cortex**	Cr	2.29 (0.39)	2.67 (0.67)	3.22	1.19	0.232
	GABA	0.47 (0.37)	0.64 (0.31)	29.41	1.49	0.136
	GSH	0.64 (0.06)	0.67 (0.19)	2.37	0.29	0.772
	Gln	0.70 (0.32)	0.92 (0.27)	15.61	1.42	0.157
	Glu	3.20 (0.34)	3.39 (0.55)	2.19	0.75	0.451
	Ins	3.46 (0.37)	3.76 (0.43)	7.28	1.35	0.176
	NAA	4.33 (0.59)	4.58 (0.51)	2.7	0.93	0.353
	NAAG	0.24 (0.15)	0.27 (0.15)	2.19	0.22	0.824
	PCh	1.00 (0.18)	1.01 (0.17)	1.79	0.19	0.851
	PCr	2.75 (0.59)	2.39 (0.73)	0.45	−1.00	0.315
**Brainstem**	Cr	3.43 (1.37)	3.00 (1.48)	0.97	−0.09	0.928
	GABA	1.54 (0.91)	1.56 (0.98)	0.93	−0.13	0.898
	GSH	0.75 (0.30)	0.86 (0.50)	0.88	−0.12	0.901
	Gln	0.94 (0.93)	0.99 (0.91)	0.87	−0.25	0.799
	Glu	3.06 (1.17)	3.22 (1.37)	1.1	0.25	0.801
	Ins	5.31 (1.48)	5.12 (1.26)	0.88	−0.3	0.764
	NAA	6.64 (5.95)	1.04 (0.94)	0.39	−1.32	0.186
	NAAG	1.45 (0.94)	2.04 (0.65)	3.7	1.51	0.131
	PCh	2.32 (0.19)	2.33 (0.37)	1.07	0.04	0.965
	PCr	3.74 (1.70)	3.41 (1.59)	0.96	−0.16	0.872
**Dorsolateral prefrontal cortex**	Cr	2.43 (0.98)	2.54 (0.89)	1.16	0.25	0.803
	GABA	0.89 (0.38)	0.82 (0.44)	0.63	−0.38	0.702
	GSH	0.44 (0.37)	0.73 (0.15)	23.11	1.65	0.100
	Gln	0.89 (0.30)	0.88 (0.45)	2.41	0.72	0.469
	Glu	3.19 (0.79)	3.20 (0.77)	1.02	0.03	0.976
	Ins	3.42 (0.78)	3.48 (0.55)	1.19	0.2	0.840
	NAA	4.87 (0.70)	5.60 (0.49)	8.49	2.03	0.042
	NAAG	0.49 (0.26)	0.38 (0.20)	0.04	−1.22	0.224
	PCh	0.86 (0.19)	0.90 (0.21)	2.68	0.37	0.713
	PCr	2.31 (1.06)	2.70 (0.81)	1.76	0.9	0.368

Note: Cr, creatine; GABA, gamma-aminobutyric acid; GSH, glutathione; Gln, glutamine; Glu, glutamate; NAA, N-acetyl aspartate; NAAG, N-acetyl aspartyl glutamate; Ins, myo-inositol; PCh, phosphocholine; PCr, phosphocreatine; metabolite data are presented as millimole per liter. The threshold of significance with Bonferroni correction for the set of all comparisons was 0.005.

**Table 3:** Comparisons of performance parameters between participants with myalgic encephalomyelitis/chronic fatigue syndrome (ME/CFS) and healthy controls (HCs) during fMRI scans with n-back task conditions.

**Reaction time (s)**				
	Task difficulty	1-back (Mean ± SD)	2-back (Mean ± SD)	Main Effect (Task)
Group	HCs	0.58 ± 0.13	0.65 ± 0.16	rmANOVA:
	ME	0.63 ± 0.14	0.68 ± 0.15	F(1, 21) = 12.61; *p* < 0.001
Main Effect (Group)		rmANOVA: F (1, 21) = 0.17; *p* = 0.69		Interaction (Task-Group)
				rmANOVA:
				F(2, 21) = 1.24; *p* = 0.280
**Accuracy (%)**				
	Task difficulty	1-back (Mean±SD)	2-back (Mean±SD)	Main Effect (Task)
Group	HCs	89.17 ± 12.32	85.00 ± 13.14	Wilcoxon Signed-Ranks:
	ME	92.48 ± 13.14	89.82 ± 14.10	Z = −2.18; *p* = 0.030
Main Effect (Group)		Mann–Whitney U: Z = −0.45; *p* = 0.69		Interaction (Task-Group)
				n/a

Note: rmANOVA, repeated measures analysis of variance; SD, standard deviation; n/a, not applicable; s, second.

**Table 4:** Correlation coefficients between featured assessments.

	r, Spearman’s Correlation Coefficient with *p* Values	Quality of Sleep	Fatigue Severity Scale	Visual Analog Fatigue Scale	Visual Analog Pain Scale	Generalized Anxiety Disorder 2-Item	Patient Health Questionnaire-2
**L-DLPFC-NAA**	r	0.64	0.52	0.54	0.43	0.42	0.25
	*p*	0.004 *	0.040 *	0.036 *	0.117	0.192	0.257
**Average Hand Grip Strength**	r	−0.50	0.01	−0.32	−0.55	−0.33	−0.37
	*p*	0.048 *	0.962	0.152	0.028 *	0.122	0.085
**Number of Voxels Activated: 1 Back**	r	0.15	0.31	0.03	0.42	0.02	0.01
	*p*	0.503	0.148	0.895	0.094	0.918	0.959
**Number of Voxels Activated: 2 Back**	r	−0.13	0.07	−0.06	−0.1	−0.01	0.29
	*p*	0.561	0.759	0.804	0.66	0.948	0.181

Note: L-DLPFC-NAA left-dorsolateral prefrontal cortex N-acetyl aspartate. r, Spearman’s correlation coefficient; *p*, level of significance, * indicates a significant difference was observed at *p* < 0.050, false discovery rate corrected.

## Data Availability

Data supporting the reported results may be available on request for research purposes with a formal request emailed to the corresponding authors and project principal investigators and permission for data access for secondary analysis will be provided upon research ethics approval.

## Supplementary Materials

The following supporting information can be downloaded at: https://www.mdpi.com/article/10.3390/medicina60081370/s1, Table S1: Functional MRI activation contrasting ME/CFS and HC groups; Table S2: List of abbreviations ordered alphabetically.
