# Supplementary material for: A Multimodal Magnetic Resonance Imaging Study on Myalgic Encephalomyelitis/Chronic Fatigue Syndrome: Feasibility and Clinical Correlation
Source: Medicina (Kaunas). 2024 Aug 22;60(8):1370. doi: 10.3390/medicina60081370 (PMC11356663; doi:10.3390/medicina60081370)
Supplement: Supplementary file 1 [file medicina-60-01370-s001.zip › medicina-3128631-supplementary.pdf]

medicina MDPI

Supplementary Materials

Table S1: Functional MRI activation contrasting ME/CFS and HC groups

Contrast	Number of Voxels	Max Z-value	p-value	Coordinates for Local Maxima			Side, Major Lobe	Cortical Structures [a]
				x *(mm)*	*y* *(mm)*	*z* *(mm)*		
HC > ME (1-Back)	2081	9.28	<0.001	-39	24	46	Left, Frontal	81% Middle Frontal Gyrus
	528	5.56	0.006	-46	24	-2	Left, Frontal	38% Inferior Frontal Gyrus [pars triangularis]<br>26% Frontal Operculum Cortex<br>7% Frontal Orbital Cortex
	553	6.85	0.005	51	8	53	Right, Frontal	11% Middle Frontal Gyrus
	1422	7.8	<0.001	-4	-58	56	Left, Occipital	84% Precuneous cortex
	1201	7.56	<0.001	-46	-66	12	Left, Occipital	67% Lateral Occipital Cortex<br>8% Middle Temporal Gyrus [Temporooccipital]
	356	4.84	0.029	64	-32	46	Right, Parietal	44% Supramarginal Gyrus
	325	6.67	0.04	24	-76	48	Right, Occipital	64% Superior Lateral Occipital Cortex
ME > HC (1-Back)	45356	9.61	<0.001	-14	54	-22	Left, Frontal	***64% Frontal Pole***
	351	6.91	0.031	-58	16	14	Left, Frontal	74% Inferior Frontal Gyrus [pars opercularis],<br>5% Precentral Gyrus
	618	7.64	0.003	-44	16	34	Left, Temporal	46% Middle Frontal Gyrus<br>8% Inferior Frontal Gyrus [pars opercularis]
	339	6.71	0.0345	1	20	30	Right, Frontal	***86% Anterior Cingulate Gyrus***
	280	6.71	0.035	16	24	-22	Right, Frontal	81% Frontal Orbital Cortex
	848	7.9	<0.001	50	-72	-6	Right, Occipital	74% Lateral Occipital Cortex [inferior division]
	339	6.71	0.035	5	-23	-22	Right, Brain Stem	***74% Brain Stem***

**HC > ME (2-Back)**	504	5.96	0.007	52	0	54	Left, Frontal	17% Precentral Gyrus
	347	6.08	0.031	-62	-37	40	Left, Parietal	78% Supramarginal Gyrus
	1380	7.56	<0.001	-40	-60	4	Left, Occipital	12% Lateral Occipital Cortex [inferior division] 11% Middle Temporal Gyrus [temporooccipital]
	445	6.4	0.012	-16	-70	66	Left, Occipital	34% Lateral Occipital Cortex [superior division]
	735	6.54	0.001	60	-26	34	Right, Parietal	49% Supramarginal Gyrus [anterior division] 7% Parietal Operculum Cortex 5% Postcentral Gyrus
**ME > HC (2-Back)**	6815	7.36	<0.001	30	64	-10	Left, Frontal	***67% Frontal Pole***
	3861	6.65	<0.001	16	32	54	Left, Frontal	67% Superior Frontal Gyrus, ***9% Frontal Pole,***
	673	5.6	0.002	-42	10	54	Left, Frontal	71% Middle Frontal Gyrus
	1442	6.11	<0.001	-64	-12	30	Left, M Parietal	84% Postcentral Gyrus
	461	5.78	0.01	-50	-40	-2	Left, Occipital	32% Middle Temporal Gyrus [posterior division], 9% Middle Temporal Gyrus [temporooccipital]
	513	5.81	0.0061	-8	-22	-16	Left, Brain Stem	*13% Brain Stem*
	482	5.4	0.0081	54	-22	52	Right, M Parietal	64% Postcentral Gyrus, 12% Supramarginal Gyrus [anterior division]
	1996	8.17	<0.001	51	-40	51	Right, M Parietal	58% Supramarginal Gyrus [posterior division], 13% Angular Gyrus
	7018	8.28	<0.001	3	-67	50	Right, Occipital	90% Precuneous Cortex
	511	5.51	0.006	61	-35	-12	Right, Occipital	59% Middle Temporal Gyrus [posterior], 10% Middle Temporal Gyrus [temporooccipital]

1027	7.74	<0.001	54	-72	0	Right,Occipital	78% Lateral Occipital Cortex [inferior division]
516	5.71	0.006	46	-52	-42	Right	Cerebellum

Note [a]: The percentage values show the probability of the centre of the cluster being located in named structures using Harvard-Oxford cortical and subcortical structural atlas [33].

Table S2: List of abbreviations ordered alphabetically.

ACC	Anterior Cingulate Cortex
ANOVA	Analysis of Variance
BS	Brainstem
Cr	Creatine
CRLB	Cramér-Rao Lower Bounds
CV	Coefficient of Variance
EPI	Echo Planar Imaging
fMRI	functional Magnetic Resonance Imaging
FOV	Field of View
FSL	Functional Magnetic Resonance Imaging of the Brain Software Library
FSS	Fatigue Severity Scale
GABA	Gamma-Aminobutyric Acid
GAD-2	Generalized Anxiety Disorder 2-item scale
GLM	General Linear Model
GLN	Glutamine
GLU	Glutamate
GRE	Gradient Echo
GPC	Glycerophosphorylcholine
GSH	Glutathione
HC	Healthy control
HGS	Hand Grip Strength
LCModel	Linear Combination of Model
L-DLPFC	Left Dorsolateral Prefrontal Cortex
LAC	Lactate
ME/CFS	Myalgic Encephalomyelitis / Chronic Fatigue Syndrome

MNI152	Montreal Neurological Institute's template of 152 averaged brain images
MRI	Magnetic Resonance Imaging
MRS	Magnetic Resonance Spectroscopy
MNI	Montreal Neurological Institute
NAAG	N-Acetylaspartylglutamate
NAA	N-Acetylaspartate
OR	Odds Ratio
PCR	Phosphocreatine
PHQ-2	Patient Health Questionnaire-2
PCh	Phosphorylcholine
QOS	Quality of sleep
REDCap	Research Electronic Data Capture
RF	Radio Frequency
RT	Reaction Time
SFU	Simon Fraser University
SD	Standard Deviation
SV	Single Voxel
T1WI	T1 weighted image
T2WI	T2 weighted image
TE	Echo Time
TR	Repetition Time/
VAS	Visual Analog Scale
VAPS	Visual Analog Pain Scale
VOI	Voxel of Interest
